# Oxidative Stress Markers and Their Dynamic Changes in Patients after Acute Ischemic Stroke

**DOI:** 10.1155/2016/9761697

**Published:** 2016-09-28

**Authors:** Ingrid Žitňanová, Pavol Šiarnik, Branislav Kollár, Mária Chomová, Petra Pazderová, Lucia Andrezálová, Miriam Ježovičová, Katarína Koňariková, Lucia Laubertová, Zuzana Krivošíková, Laura Slezáková, Peter Turčáni

**Affiliations:** ^1^Institute of Medical Chemistry, Biochemistry and Clinical Biochemistry, Faculty of Medicine, Comenius University, Bratislava, Slovakia; ^2^1st Department of Neurology, Faculty of Medicine, Comenius University, Bratislava, Slovakia; ^3^2nd Department of Internal Medicine, Faculty of Medicine, Comenius University, Bratislava, Slovakia

## Abstract

We have focused on determining the range of oxidative stress biomarkers and their dynamic changes in patients at different time points after the acute ischemic stroke (AIS). 82 patients with AIS were involved in our study and were tested: within 24 h from the onset of the attack (group A); at 7-day follow-up (group B); and at 3-month follow-up (group C). 81 gender and age matched volunteers were used as controls. Stroke patients in group A had significantly higher concentrations of plasma lipid peroxides and urine 8-isoprostanes when compared with controls. Protein carbonyls were not significantly different in any experimental group compared to controls. Antioxidant capacity of plasma was increased only in experimental group C. Activities of superoxide dismutase and catalase were elevated in all three experimental AIS groups compared to controls. Paraoxonase activity was reduced in groups A and B and unchanged in group C when compared to controls. Glutathione peroxide activity was elevated only in group A. Our results suggest that free radical damage is the highest within 24 h after the attack. During the next 3 months oxidative damage to lipids caused by free radicals is reduced due to activated antioxidant system.

## 1. Introduction

Stroke is a serious health, social, and economic problem of society. It is the third leading cause of death, after tumor disease and myocardial infarction, and the first cause of disability in patients in western world [1]. Reactive oxygen species (ROS) produced during ischemic and reperfusion phases in acute ischemic stroke (AIS) can lead to brain injury by attacking cerebral vasculature. They can damage macromolecules in cellular components such as cellular proteins, membrane lipids, and nucleic acids [2, 3]. Brain tissue is especially vulnerable to ROS due to low levels of endogenous antioxidant enzymes such as glutathione peroxidase and catalase [4]. Moreover, the brain is rich in iron which can activate generation of hydroxyl radical by Fenton reaction. It is also rich in polyunsaturated fatty acids which are prone to peroxidation during increased production of ROS [1]. Main sources of ROS in cerebral ischemia are mitochondria, NADPH oxidase, phospholipase A2, and cyclooxygenase [5].

Oxidative stress has been defined as an imbalance between oxidants and antioxidants in favor of oxidants, potentially leading to damage to lipids, proteins, and nucleic acids [6]. Oxidative stress is thought to play a key role in pathogenesis of acute ischemic stroke. Reactive oxygen species produced during ischemic and reperfusion phases of ischemic stroke can attack cerebral tissue. Several metabolites generated during these processes can be detected in the organism. To estimate oxidative damage to lipids malondialdehyde, hydroxynonenal, lipid peroxides, and isoprostanes have been used as markers [3, 7]. To estimate oxidative damage to proteins, protein carbonyls or 3-nitrotyrosine have been detected as markers [8]. However, there is a lack of studies on protein carbonyls in acute ischemic stroke. Moreover, there is scarce information on dynamic changes of the markers of oxidative stress after the stroke. Most of the studies are cross-sectional or have short follow-up periods after the stroke. Therefore, we have focused on determining the range of oxidative stress biomarkers and antioxidants in stroke patients over a 3-month period after acute ischemic stroke.

## 2. Material and Methods

### 2.1. Study Population

82 consecutive patients (mean age 68.70 ± 15.90 years) (46 males and 36 females) with acute ischemic stroke (AIS) as well as 81 age matched (64.91 ± 9.01 years) control individuals (36 males and 45 females) were included in this study. To assess dynamic changes of examined parameters, blood and urine were collected from patients at the start of this study and reexamined at 7-day and 3-month follow-ups. All study participants were admitted to the University Hospital in Bratislava, Slovakia, between March 2014 and February 2015. To determine the subtype of stroke, clinical examination followed by a CT scan of the brain was done. Participants involved in our project as controls have never experienced any stroke attacks and were without any acute or chronic diseases.

Data on acute stroke admissions were collected at the 1st Department of Neurology in Bratislava, Slovakia, by trained professionals. These data included routine haematological and biochemical parameters, as well as vascular risk factors such as age, sex, diabetes mellitus, and ischemic heart disease. Additional variables collected included any current treatment with antihypertensives, diabetic medications, or cholesterol-reducing medications. The National Institute of Health Stroke Scale (NIHSS) scoring system was used to evaluate the severity of outcome and the modified Ranking Scale (mRS) to assess “global disability” with a focus on mobility [9]. This study was approved by the local ethics committee. All participants in our study signed an informed consent.

### 2.2. Blood Samples

Fasting venous blood with/without EDTA was collected from each patient within 24 h after AIS (group A) and then at 7-day (group B) and 3-month (group C) follow-ups. From control individuals blood was collected only once after overnight fasting (group Co).

### 2.3. Plasma, Hemolysate, Serum, and Urine Samples

To obtain plasma, blood samples with EDTA were centrifuged for 5 min at 1200 ×g and at 4°C, aliquoted, stored at −80°C, and used for determination of oxidative stress parameters. Erythrocytes (0.5 mL) were washed three times with physiological solution (5 mL 0.9% NaCl) and centrifuged at 660 ×g for 5 min at 4°C with subsequent hemolysis in chilled distilled water. Hemolysates were stored at −80°C and used for determination of hemoglobin concentration and activities and protein expression of antioxidant enzymes.

Serum was collected from blood samples without EDTA. Samples were centrifuged for 5 min at 1200 ×g and at 4°C and serum was used for PON1 activity and lipid profile determination. Urine samples were stored in aliquots at −80°C.

### 2.4. Examined Parameters

Total cholesterol (TC), HDL-cholesterol, LDL-cholesterol, triacylglycerols (TAG) in serum, and creatinine in urine were determined in the certified laboratory. Hemoglobin concentration was determined in the lysates of erythrocytes using Drabkin's reagent [10]. The presence of lipid peroxides was measured in plasma samples spectrophotometrically (UV-1800 Shimadzu Spectrophotometer) according to assay by El-Saadani et al. [11]. Isoprostanes (8-*iso* prostaglandin F_2*α*_) in urine were determined by the commercial EIA kit (Cayman Chemical, USA) following manufacturer's instructions. Protein carbonyls in plasma were detected by the commercial OxiSelectTM protein carbonyl ELISA kit (Cell Biolabs, USA) following the manufacturer's instructions. Plasma antioxidant capacity was measured by the TEAC assay [12]. Trolox, a synthetic and water soluble form of vitamin E, was used as a reference antioxidant. Paraoxonase activity (PON1) in serum was determined spectrophotometrically using phenylacetate as a substrate [13]. The molar extinction coefficient 1310 mol^−1^·L·cm^−1^ was used to express enzyme activity (U/mL). 1 U is defined as 1 *μ*mol of phenol produced per one minute. To determine the SOD activity in lysates of erythrocytes the SOD Assay kit (Sigma-Aldrich Co., USA) was employed. SOD activity was expressed in U/mg Hb where 1 U of SOD activity is defined as the amount of SOD required to inhibit the rate of chromagen reduction by 50%. Catalase activity in erythrocytes was determined according to Bergmeyer [14]. GPx activity in hemolysates was determined by the commercial kit (Cayman Chemical, USA) according to manufacturer's protocol. Each sample was analyzed in triplicate.

### 2.5. Protein Expression of Antioxidant Enzymes

Expression of SOD and catalase was determined by the SDS-PAGE and semiquantitative western blot analysis. Samples of erythrocyte lysates (20 *μ*g protein) were separated by SDS-PAGE on 6–15% gradient polyacrylamide gels for determination of SOD1 and catalase protein levels. Separated proteins were transferred to PVDF membranes (Millipore Corp., USA) using semidry Trans-Blot Turbo Blotting System (BioRad, USA). The membranes were probed with primary antibodies specific for SOD1 (1 : 500, Santa Cruz Biotechnology, USA) and catalase (1 : 2000, Millipore Corp., USA). A peroxidase-linked antirabbit IgG (1 : 10 000, Santa Cruz Biotechnology, USA) was used as the secondary antibody. Immunoreactive proteins were visualised by Clarity Western ECL Chemiluminescent Substrate (BioRad, USA), checked for the protein load and band densities, and analyzed on ChemiDoc MP imaging system (BioRad, USA).

### 2.6. Statistical Analysis

The statistical analyses were performed using SPSS version 18 (SPSS Inc., USA). Significance level was set at *P* < 0.05. Variables were expressed as means ± standard error of mean or standard deviation. In the case of not normally distributed data median was used with interquartile range (IQR), minimal and maximal values. To compare groups, Student's *t*-test and Mann–Whitney* U* test were used for particular variables. Pearson or Spearman correlation coefficients were used to determine relationships among particular parameters. Results from western blot analysis were evaluated by one-way ANOVA, followed by Tukey's test to determine differences between individual groups.

## 3. Results

As shown in Table 1, HDL-cholesterol, LDL-cholesterol, and total cholesterol levels were significantly lower in the groups after AIS when compared to the control group. Higher HDL-cholesterol in control individuals might serve as a protecting parameter against AIS.

### 3.1. Markers of Oxidative Damage to Lipids and Proteins

Plasma lipid peroxide levels were increased in ischemic stroke group (A) when compared to those of healthy controls (Co). At 7-day and 3-month follow-ups (B and C) levels of lipid peroxides dropped to the level of healthy controls. Also urine isoprostane levels (the marker of oxidative damage to lipids) were significantly elevated in patients examined within 24 h after the AIS (A). Plasma protein carbonyl levels were not significantly changed in all experimental groups (Table 2).

### 3.2. Total Antioxidant Capacity of Plasma

Total antioxidant capacity (TEAC) of plasma was significantly increased only in patients examined 3 months after the onset of AIS (C). All other experimental groups had plasma antioxidant capacity similar to healthy controls (Table 2).

### 3.3. Superoxide Dismutase (SOD)

**Figure 1:**
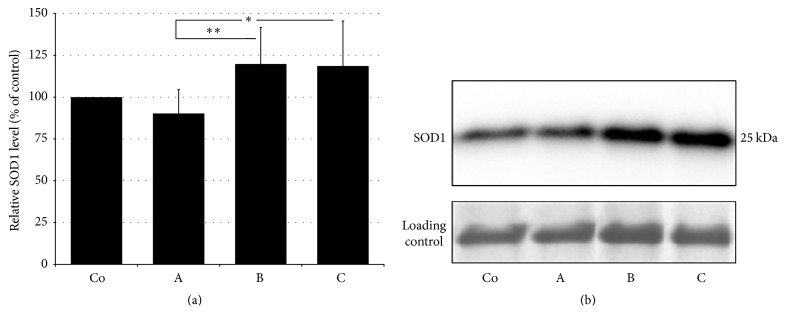
Semiquantitative evaluation (a) and representative blot of SOD1 protein expression (b) in lysates of erythrocytes in controls (Co) and ischemic stroke patients. A: patients within 24 h after AIS, B: patients at 7-day follow-up, and C: patients at 3-month follow-up. Data are presented as the means ± SD. *n* = 6; ^*∗*^ *P* < 0.05; ^*∗∗*^ *P* < 0.01 versus acute ischemic stroke group A.

SOD activity in hemolysates was significantly elevated in stroke patients in all experimental groups, reaching the highest activities in group B (Table 2). Using western blot analysis we have examined expression of SOD in lysates of erythrocytes and detected significantly increased expression in experimental groups B and C (Figures 1(a) and 1(b)). These elevated activities of SOD signify an adaptive response of organism as well as restored protein synthesis after AIS, since western blot analysis of erythrocyte lysates revealed significantly increased protein levels in the B and C experimental groups compared to acute ischemic stroke group (A).

### 3.4. Catalase

**Figure 2:**
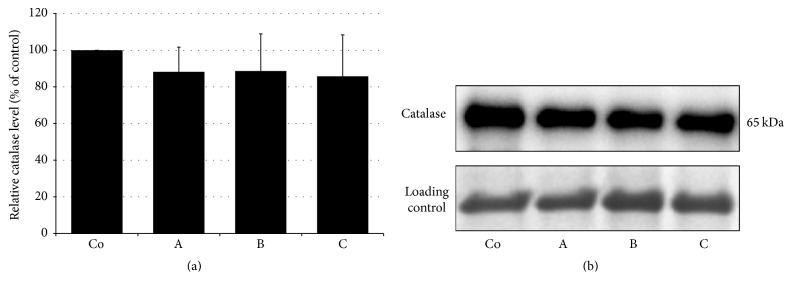
Semiquantitative evaluation (a) and a representative blot of catalase protein expression (b) in lysates of erythrocytes in controls (Co) and ischemic stroke patients. A: patients within 24 h after AIS, B: patients at 7-day follow-up, and C: patients at 3-month follow-up.

All stroke patients (groups A–C) had catalase activities significantly elevated compared to the controls. The highest values were reached in the experimental group B (Table 2). Western blot analysis revealed unchanged catalase expression in all study groups (Figures 2(a) and 2(b)).

### 3.5. Glutathione Peroxidase

Activities of GPx were significantly increased (*P* > 0.05) in AIS group A. Other experimental groups were not significantly changed compared to healthy controls (Table 2).

### 3.6. Paraoxonase

Paraoxonase (PON) activities were significantly reduced (*P* > 0.001) in AIS patients in groups A and B compared to controls and they returned to the control level after 3 months.

### 3.7. Correlations between Parameters

Significant positive and negative correlations found between measured parameters in experimental groups of AIS patients are listed in Table 3 and of controls in Table 4.

## 4. Discussion

Results of studies addressing the levels of lipid parameters in AIS patients are quite inconsistent. Our results are in agreement with several studies [15] indicating a lipid lowering effect of acute ischemic stroke. The mechanism of this effect is unclear. It might be associated with stress and overproduction of catecholamines leading to the reduction of serum cholesterol [16]. Serum HDL-cholesterol, LDL-cholesterol, and total cholesterol as well as triacylglycerols levels were found to be reduced initially, but at 3 months all values increased.

In this study, we have found elevated levels of a marker of oxidative damage to lipids, plasma lipid peroxides in stroke patients (group A) when compared with controls. Surprisingly, this increase of lipid peroxides was inversely proportional to the patient's age. Similar phenomenon has been reported also by Halper et al. [17] who found the change from a positive correlation between age and oxidative stress/DNA instability to the negative associations in people after 60 to 70 years of age. Individuals in our study (mean age 68.7 years) belong to the same age range.

Increased levels of lipid peroxides in patients after AIS have also been reported by other authors [18, 19]. Also, another marker of lipid oxidation, urine 8-isoprostanes, was significantly elevated in acute stroke patients (group A). Later evaluations (groups B and C) showed 8-isoprostane levels similar to those of the control group (Co). 8-Isoprostanes are formed by nonenzymatic free radical-catalyzed peroxidation of arachidonic acid present in phospholipids and may reflect oxidative stress in clinical conditions. Hoffman et al. [20] have shown that 8-isoprostanes are formed after brain injury and are generated mostly within 24 h after the attack, which correlates with our results. Published data indicate that isoprostanes can also have potent vasoconstriction effects on the peripheral vasculature, contributing to the increased sensitivity of the brain to oxidative stress and to the vascular pathobiology [21].

A marker of oxidative damage to proteins (protein carbonyls) was unchanged in all experimental groups. There are only a few studies on protein oxidation in patients after AIS which claim unchanged [22] or increased [23] generation of protein carbonyls. We have found a strong negative correlation between the plasma protein carbonyls and total antioxidant capacity (TEAC) only in the control group.

In addition to increased plasma lipid peroxide and urinary 8-isoprostane levels in stroke patients (group A), we have also detected significantly increased activities of antioxidant enzymes, SOD and catalase in lysates of erythrocytes. The elevated SOD activity can be related to the increased production of ROS during AIS represented by increased oxidative damage to lipids. It is known that ischemic attack results in inhibition of proteosynthesis. Restoration of blood flow and energy homeostasis results in restoration of proteosynthesis [24]. Therefore, the increased antioxidant activity of the enzyme in patients' groups might not only respond to the increased production of ROS and the potentiation of the antioxidant defenses, but also be a consequence of the reestablishment of the proteosynthesis. This assumption is supported by our results of western blot analysis, which revealed an increased SOD protein expression at later time points when compared to group A. This reaction may also represent an adaptive response of the organism (i.e., ischemic preconditioning) to the pathological event at the system level [25]. Results on SOD activities in stroke patients in different studies are inconsistent. Several studies have reported reduced [26] or unchanged [27] SOD activities in lysates of erythrocytes. The increased production of superoxide radical usually occurs during reperfusion period in stroke patients [28]. Elevated SOD activities result in increased production of hydrogen peroxide which can stimulate catalase activity. Increased catalase activity also supports the existence of oxidative stress. It appears, however, that at the level of proteosynthesis and the adaptive response of the body to ischemic-reperfusion “experience” catalase is not as susceptible, in view of the steady state of the enzyme protein expression in all experimental groups. Therefore, catalase might not participate in “ischemic preconditioning” of the body in view of its sufficient antioxidant capacity and SOD.

Presence of oxidative stress in patients with AIS has also been supported by reduced PON1 activities in experimental groups A and B. Our results are consistent with results of other studies [29]. Reduced PON1 activities are assumed to increase the risk of atherosclerosis which is a stroke risk factor [30]. There was a strong negative correlation reported between HDL-cholesterol lipoproteins and the development of atherosclerosis [31]. Paraoxonase is the enzyme associated with HDL-cholesterol lipoprotein particles and can prevent oxidation of LDL lipoproteins. This association has been confirmed by finding strong positive correlations between paraoxonase activity and HDL-lipoprotein levels in the control group and patient groups A and B. In the control group, paraoxonase activity positively correlated with plasma total antioxidant capacity (TEAC). However, activity of this antioxidant enzyme is reduced with the age in both the control group and group A of patients with AIS.

## 5. Conclusions

This study is one of the most complex works examining oxidative stress in patients after AIS. Most studies either are cross-sectional or have short follow-up periods after stroke. Our study records the dynamics of changes in parameters of oxidative stress for a relatively long time period—three months after the ischemic episode.

Our study reports increased lipid oxidation and increased activities of SOD and catalase in patients after AIS. Increased antioxidant enzyme activities might reduce the damage induced by free radicals which is reflected in reduced levels of lipid peroxides at 7-day and 3-month follow-ups and provide protection from neurological damage. Activities of antioxidant enzymes remained elevated even at 3 months after AIS.

## Figures and Tables

**Table 1:** Characteristics of lipid profile in serum of controls and patients after AIS.

Parameter	Co	A	B	C
HDL-C (mmol/L)	1.28 ± 0.3	1.15 ± 0.3^*∗∗*^	0.93 ± 0.24^*∗∗∗*^	1.13 ± 0.38^*∗*^
LDL-C (mmol/L)	3.61 ± 1.11	3.18 ± 1.11^*∗*^	2.64 ± 0.61^*∗∗*^	2.86 ± 1.58^*∗∗*^
TC (mmol/L)	5.09 ± 1.21	4.43 ± 1.18^*∗∗∗*^	3.64 ± 0.94^*∗∗∗*^	4.12 ± 1.12^*∗∗∗*^
TAG (mmol/L)	1.48	1.09	1.17	1.39
	(1.14–2.08)	(0.80–1.67)	(0.82–1.56)	(0.84–1.74)

Data are presented as means ± SD or median with an interquartile range (Q1–Q3, 25–75%). Co: control individuals; A: patients within 24 h after AIS, B: patients at 7-day follow-up, and C: patients at 3-month follow-up; HDL-C: high density lipoprotein cholesterol; LDL-C: low density lipoprotein cholesterol; TC: total cholesterol; TAG: triacylglycerols. ^*∗*^ *P* < 0.05; ^*∗∗*^ *P* < 0.01; ^*∗∗∗*^ *P* < 0.001 versus controls.

**Table 2:** Levels of oxidative stress markers and activities of antioxidant enzymes in experimental groups of ischemic stroke patients (A, B, C) and healthy controls.

Parameter (units)	Co	A	B	C
Lipid peroxides in plasma (nmol/mL)	43.48 ± 2.98	55.09 ± 3.55^*∗*^	43.62 ± 3.72	44.37 ± 5.47
8-Isoprostanes in urine (ng/mmol creatinin)	32.57 ± 4.40	68.69 ± 10.93^*∗∗∗*^	43.72 ± 8.65	43.89 ± 12.70
Protein carbonyls in plasma (nmol/mg protein)	0.25 ± 0.04	0.22 ± 0.03	0.28 ± 0.04	0.20 ± 0.07
TEAC in plasma (mmol/L)	1.49 ± 0.13	1.64 ± 0.12	1.47 ± 0.17	2.77 ± 0.17^*∗∗∗*^
PON1 in serum (U/mL)	104.07 ± 3.04	86.45 ± 3.57^*∗∗∗*^	85.86 ± 4.61^*∗∗∗*^	102.39 ± 7.95
SOD in hemolysates (U/mg Hb)	661.44 ± 7.15	697.62 ± 8.95^*∗∗*^	750.45 ± 9.72^*∗∗∗*^	747.41 ± 17.27^*∗∗∗*^
Catalase in hemolysates (*µ*kat/g Hb)	3.61 ± 0.17	4.92 ± 0.17^*∗∗∗*^	6.56 ± 0.24^*∗∗∗*^	4.85 ± 0.21^*∗∗∗*^
GPx in hemolysates (U/g Hb)	24.83 ± 1.24	36.16 ± 4.91^*∗*^	26.28 ± 1.51	23.55 ± 2.60

Data are presented as means ± SEM. Co: control individuals; A: patients within 24 h after AIS, B: patients at 7-day follow-up, and C: patients at 3-month follow-up; TEAC: trolox equivalent antioxidant capacity of plasma; PON1: paraoxonase activity; SOD: superoxide dismutase activity; GPx: glutathione peroxidase activity; Hb: hemoglobin. ^*∗*^ *P* < 0.05; ^*∗∗*^ *P* < 0.01; ^*∗∗∗*^ *P* < 0.001 versus controls.

**Table 3:** Spearman correlations among measured parameters in stroke patients.

	Group		*r*	*P*
PON1	A	Age	−0.325	0.011
		Total cholesterol	0.328	0.009
		HDL	0.305	0.016
	B	Total cholesterol	0.475	0.003
		HDL	0.429	0.005
	C	Total cholesterol	0.587	0.017
		Lipid peroxides	0.636	0.048

SOD	C	Lipid peroxides	−0.875	0.001
		PON1	0.636	0.048
		LDL	0.9	0.037

Catalase	A	LDL	0.273	0.029

Lipid peroxides	A	Age	−0.281	0.015

Spearman correlation coefficients (*r*) are indicated; *P* < 0.05 is considered statistically significant. Group A: patients within 24 h after AIS; group B: patients at 7-day follow-up; and group C: patients at 3-month follow-up. PON1: paraoxonase; SOD: superoxide dismutase.

**Table 4:** Spearman correlations among measured parameters in controls.

	Parameter	*r*	*P*
PON1	TEAC	0.249	0.042
	HDL-cholesterol	0.315	0.007
	Age	−0.314	0.008

TEAC	Protein carbonyls	−0.416	0.0003

TAG	Lipid peroxides	0.351	0.0025

Spearman correlation coefficients (*r*) are indicated; *P* < 0.05 is considered statistically significant. PON1: paraoxonase; TEAC: trolox equivalent antioxidant capacity in plasma; TAG: triacylglycerols.

## Abbreviations

AIS: Acute ischemic stroke
ROS: Reactive oxygen species
SOD: Superoxide dismutase
GPx: Glutathione peroxidase
PON: Paraoxonase
NIHSS: National Institute of Health Stroke Scale
mRS: modified Ranking Scale
TC: Total cholesterol
TAG: Triacylglycerols.
